# Incaspitolide A extracted from *Carpesium cernuum* induces apoptosis *in vitro* via the PI3K/AKT pathway in benign prostatic hyperplasia

**DOI:** 10.1042/BSR20210477

**Published:** 2021-06-21

**Authors:** Xiaoyue Chen, Jingrui Song, Dongbo Yuan, Qing Rao, Kehua Jiang, Shuhui Feng, Guohua Zhu, Chen Yan, Yanmei Li, Jianguo Zhu

**Affiliations:** 1 Department of Biomedicine, Guizhou University, Guiyang 550002, P.R. China; 2 State Key Laboratory of Functions and Applications of Medicinal Plants, Guizhou Medical University, Guiyang 550014, China; 3 Department of Urology, Guizhou Provincial People’s Hospital, Guiyang 550002, P.R. China; 4 Department of Pharmacy, An Shun City People’s Hospital, Anshun 561000, P.R. China

**Keywords:** apoptosis, benign prostatic hyperplasia, Carpesium cernuum, cell cycle, Incaspitolide A, PI3K/AKT pathway

## Abstract

Benign prostatic hyperplasia (BPH) is a common disease that occurs mainly in older men. The pathogenesis of BPH is complex and patients face a prolonged treatment course, and novel drugs with better selectivity and lower toxicity are required. Incaspitolide A (compound TMJ-12) is a germacrane-type sesquiterpenoid compound extracted from the plant *Carpesium carnuum*. Extracts of *C. carnuum* are known to exert suppressive effects on BPH-1 cells. In the present study, we investigated the molecular mechanisms underlying the suppressive effect of TMJ-12 specifically on BPH-1 cells. A cytotoxicity assay indicated that TMJ-12 inhibited BPH-1 cell proliferation, while flow cytometry assays showed that TMJ-12 induced G_2_/M phase cell cycle arrest and the apoptosis of BPH-1 cells. TMJ-12 was also shown to regulate the expression of several apoptosis- and cell cycle-related proteins, namely Bcl-2, Bax, Bad, Caspase-9, Caspase-3, cyclin-dependent kinase 1 (CDK1), Cyclin B1, CDC25C, and c-Myc, among others. Collapse of the mitochondrial membrane potential (ΔΨm) following exposure to TMJ-12 was detected with the JC-1 staining assay. Further investigation revealed that treatment with TMJ-12 inhibited the phosphatidylinositol 3-kinase (PI3K)/protein kinase B (AKT) pathway by increasing the expression of phosphatase and tensin homolog deleted on chromosome 10 (PTEN). Taken together, the results suggest that TMJ-12 prevents BPH-1 cell proliferation via the PI3K/AKT pathway by inducing apoptosis and cell cycle arrest.

## Introduction

Benign prostatic hyperplasia (BPH) is a non-malignant enlargement of the prostate [1]. It is a common disease among older men, with up to 80% reported to have some degree of prostate enlargement by the age of 50 years [2]. The symptoms of BPH can be obstructive or irritative or may only affect the patient after micturition. Current treatments for BPH include surgery to resect the obstructive prostate tissue and minimize disease progression, or medications such as the 5-α reductase inhibitors, epristeride and finasteride, which block the conversion of testosterone into DHT, inhibiting prostatic hyperplasia, reducing prostate size, and slowing disease progression [3]. However, since most BPH patients are elderly, most of them may have serious cardiovascular and cerebrovascular complications and cannot be treated with surgery [4]. Many men are plagued with abhorrent side effects such as erectile dysfunction, ejaculatory problem, and a decrease in libido. Of note, high post-void residuals, dry mouth, constipation, and the risk of dementia limit their long-term use in the elderly [5,6]. While natural molecule compounds have milder effects and less adverse reactions, they are suitable for long-term use [7]. BPH can severely affect the health and well-being of patients, and novel drugs with better selectivity and lower toxicity are required. Drugs for the treatment of BPH mainly act by inhibiting prostate cell proliferation.

The phosphatidylinositol 3-kinase (PI3K)/protein kinase B (AKT) signaling pathway is crucial to many aspects of cell growth and survival, and the abnormal activation of the PI3K/AKT signaling pathway is associated with the occurrence and development of oncogenic effects [8]. PI3K is a heterodimer consisting of a catalytic subunit (p110) and a regulatory subunit (p85). Upon activation, PI3Ks phosphorylate phosphatidylinositol 4,5-bisphosphate [PtdIns(4,5)P_2_] to produce PtdIns(3,4,5)P_3_ [9]. AKT is the primary downstream mediator of the effects of PI3K. AKT is activated by phosphorylation at Thr^308^ by 3-phosphoinositide-dependent protein kinase 1 (PDK1) and at Ser^473^ in the hydrophobic C-terminal domain by PDK2. Abnormal levels of AKT can affect the expression of proteins regulating various cell cycle-dependent kinases, such as cyclin-dependent kinase 1 (CDK1) and CDK2, thereby affecting cell cycle regulation and causing cell cycle arrest [10,11]. Phosphorylated AKT can promote the phosphorylation of mTORC1 directly or indirectly. The mTORC1 protein complex activates the downstream eukaryotic initiation factor 4E-binding protein 1 (4EBP1) and ribosomal p70S6 kinase (p70S6K), which participate in cell cycle regulation and cell apoptosis [12,13]. The PI3K/AKT pathway can inactivate the Caspase-9 and Caspase-3 proteins, and at the same time, affect the binding of Bad and Bcl-2, thereby inhibiting cell apoptosis [14]. The phosphatase and tensin homolog deleted on chromosome 10 (PTEN), which can convert PtdIns(3,4,5)P_3_ into PtdIns(4,5)P_2_, is a negative regulator of the PI3K/AKT pathway [15,16].

**Figure 1:**
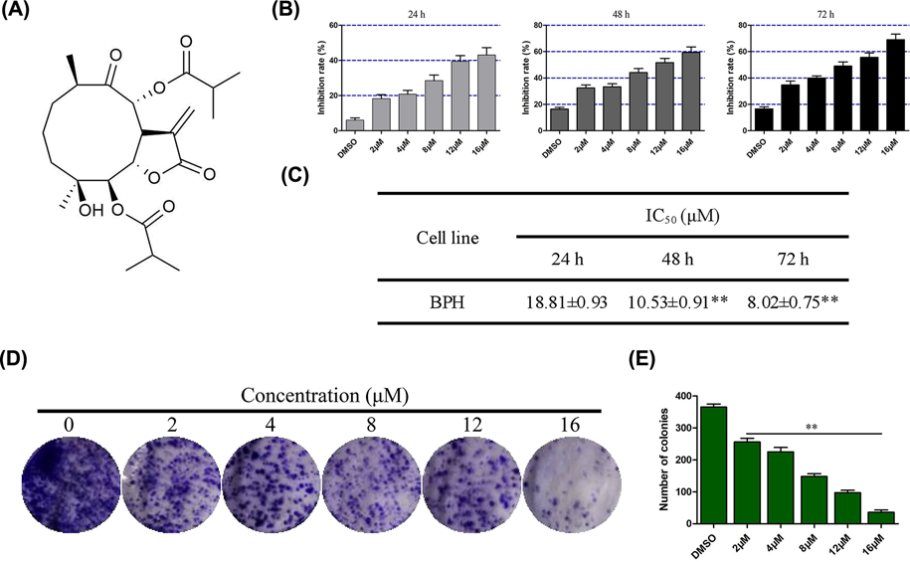
Compound TMJ-12 inhibits the proliferation of BPH-1 cells (**A**) Chemical structure of compound TMJ-12 extracted from *C. cernuum*. (**B**) BPH-1 cells were treated with various concentrations of TMJ-12 for 12, 48, or 72 h, and the effect on cell viability was determined with the MTT assay. (**C**) The IC_50_ value (μM) of TMJ-12 toward the BPH-1 cells was determined. (**D,E**) The number of colonies formed was counted and analyzed using Crystal Violet staining. Data represent the mean ± SD, *n*=3. **P<0.01 vs. the control group. Abbreviation: MTT, 3-(4,5-dimethylthiazol-2-yl)-2,5-diphenyltetrazolium bromide.

*Carpesium cernuum*, which belongs to the genus *Carpesium* (Asteraceae), is traditionally used to treat fevers, colds, bruises, and inflammatory diseases [17]. The constituents of this plant have been previously investigated and shown to contain several sesquiterpenoids with diverse bioactivities such as cytotoxicity, anti-inflammatory, and antifungal activities [18]. In recent years, sesquiterpenoids have served as a major source of lead compounds for the development of drugs targeting tumor cells [19]. In the early stage, when we checked the literature it was found that the crude drug *Carpeium cernuum* extract has the effect of inhibiting the BPH-1 cells, but the authors did not elaborate on its mechanism [20]. Therefore, our group has isolated and extracted the main component of *C. cernuum*, incaspitolide A, and conducted cytotoxicity experiments and mechanism research. The compound incaspitolide A (termed TMJ-12 in the present study, see Figure 1A) is one such sesquiterpenoid isolated from *C. cernuum* [21]. The purity analysis of TMJ-12 is described in Supplementary Data S1. In the present study, we investigated the effect of TMJ-12 on a benign prostatic hyperplasia cell line (BPH-1) and found that it induced apoptosis and cell cycle arrest by activating PI3K/AKT signaling.

## Materials and methods

### Extraction of TMJ-12

Main extraction, isolation, and purification process of TMJ-12 were described as before [21]. Incaspitolide A was obtained from fraction-5 (Fr. 5). Fr. 5 (82.0 g) was chromatographed on a silica gel column (petroleum ether–ethyl acetate from 9:1 to 5:5) to give subfraction 5e that was subjected to CC over silica gel (petroleum ether–ethyl acetate from 9:1 to 8:2) to obtain three fractions (5e-1, 5e-2, and 5e-3), 5e-3 was then separated using a Waters X-Bridge C18 column (4.6 × 250 mm) (MeCN/H_2_O = 50:50) to afford TMJ-12 (32.6 mg). Its NMR data are as follows: ^1^H-NMR (400 MHz, CDCl_3_) δ: 6.38 (1H, d, J = 1.2 Hz, H-13a), 5.92 (1H, d, J = 1.2 Hz, H-13b), 4.82 (1H, d, J = 11.2 Hz, H-6), 4.63 (1H, dd, J = 1.2, 6.0 Hz, H-9), 4.59 (1H, d, J = 6.0 Hz, H-8), 3.45 (1H, dd, J = 1.6, 11.2 Hz, H-7), 3.03 (1H, m, H-4), 1.12 (3H, s, H-14), 1.01 (3H, d, J = 6.8 Hz, H-15), ^13^C NMR (100 MHz, CDCl3) δ: 25.1 (C-1), 33.0 (C-2), 35.4 (C-3), 73.2 (C-4), 77.2 (C-5), 71.0 (C-6), 45.2 (C-7), 78.2 (C-8), 211.1 (C-9), 41.7 (C-10), 132.6 (C-11), 168.4 (C-12), 126.7 (C-13), 20.3 (C-14), 22.0 (C-15), 2 iBu (176.8, 175.1, 33.80, 33.77, 18.9, 18.8, 18.7). It was identified as incaspitolide A by comparison of the physical and spectral data with the literature [22]. Its purity was 98.65% by HPLC analysis.

### Reagents

Fetal bovine serum (FBS) was purchased from Gibco (Gaithersburg, MD, U.S.A.). Dulbecco’s modified Eagle’s medium (DMEM), 3-(4,5-dimethylthiazol-2-yl)-2,5-diphenyltetrazolium bromide (MTT), and dimethyl sulfoxide (DMSO) were purchased from Sigma–Aldrich (U.S.A.). The Annexin V-FITC Apoptosis Detection Kit and Hoechst Staining Kit were purchased from Solarbio (Beijing, China).

### Antibodies

Bax (ab32503; 1:1000), Bcl-2 (ab32124; 1:2000), CDK1 (ab201008; 1:1000), Cyclin B1 (ab32053; 1:10000), Cyclin D1 (ab40754; 1:2000), CyclinD2 (ab207604; 1:1000), c-Myc (ab32072; 1:1000), Cytochrome *c* (ab133504; 1:10000), RAS (ab52939; 1:1000), P27 (ab32034; 1:5000), P21 (ab109520; 1:1000), and PDK1 (ab202468; 1:2000) were obtained from Abcam (Cambridge, U.K.). Antibodies for Bad (9268S; 1:1000), Caspase-3 (9662S; 1:1000), cleaved Caspase-3 (9664; 1:1000), Caspase-9 (32539; 1:1000), PARP (9532; 1:1000), CDC25C (4688S; 1:1000), p-CDC25C (4901; 1:1000), PCNA (13110; 1:1000), P53 (2527S; 1:1000), PTEN (9188; 1:1000), AKT (4685; 1:1000), p-AKT (4060; 1:2000), mTOR (2983; 1:1000), p-mTOR (5536; 1:1000), EIF4E (2067; 1:1000), SAPK/JNK (9252; 1:1000), p-SAPK/JNK (9255; 1:1000), MAPK/P38 (8690; 1:1000), and p-MAPK/P38 (4511; 1:1000) were obtained from Cell Signaling Technology (Beverly, MA, U.S.A.).

### Cell culture

The BPH-1 was a gift from the Sunnybrook Research Institute (Toronto, ON, Canada). The cells were cultivated in DMEM supplemented with 10% FBS and 1% (w/v) penicillin (Solarbio) at 37°C in a CO_2_ incubator (5% CO_2_, 95% humidity). After a 24-h incubation, the cells were treated with TMJ-12 at various concentrations (2, 4, 8, 12, or 16 μM) and the cells were then collected within the specified time for further analysis.

### Cytotoxicity assay

The cytotoxicity of TMJ-12 toward the BPH-1 cell line was measured with the MTT assay according to a previously described protocol [22]. Briefly, cells were seeded in 96-well plates at a density of 1 × 10^4^ cells/well, incubated at 37°C for 24 h, and then treated with TMJ-12 (0, 2, 4, 8, 12, 16 μM) for the indicated time (24 or 48 h). Next, 20 μl of the MTT solution (5 mg/ml) was added to each well, and the plates were incubated for 4 h at 37°C. The resultant formazan crystals were dissolved in 160 μl of DMSO and the absorbance (at 490 nm) was measured using a spectrophotometer (Varioskan Flash; Thermo Fisher Scientific, Waltham, MA U.S.A.). The half-maximal inhibitory concentration (IC_50_) of TMJ-12 toward the BPH-1 cells was calculated using a cell survival curve, and a growth curve was generated based on the absorbance and concentration values.

### Colony formation assay

BPH-1 cells were seeded in six-well culture plates at a confluency of 3 × 10^5^ cells/plate, and were then treated with various concentrations of TMJ-12 (0–16 µM) for 24 h. The cells were trypsinized, resuspended in the medium, and counted. Next, the cells were re-seeded (1000 cells per medium plate) and incubated for 20 days with fresh medium added every 4 days. The cells were then fixed with 4% paraformaldehyde for 30 min and stained with Crystal Violet staining solution (Beyotime, Jiangsu, China) for 15 min before counting.

### Cell apoptosis assay and cell cycle analysis

The cell apoptosis assay and cycle analysis were performed with an FACS Calibur flow cytometer (BD Biosciences, NJ, U.S.A.). Propidium iodide (PI; Solarbio) was used for cell cycle analysis, while a staining kit containing Annexin V-fluorescein isothiocyanate (Annexin V-FITC) and PI (BD Pharmingen, San Diego, CA, U.S.A.) was used to assess cell apoptosis. BPH-1 cells (3 × 10^5^) were seeded in six-well plates for 24 h and then incubated with TMJ-12 (0, 8, or 16 µM) for 24 or 48 h. Next, the cells were washed with cold phosphate-buffered saline (PBS) and then fixed with chilled 75% ethanol overnight at −20°C. After being centrifuged at 1000 rpm for 5 min, the cells were washed with chilled PBS. For cell cycle analysis, the cells were stained with PI for 30 min at 37°C and analyzed on the flow cytometer. For the apoptosis experiment, the cells were resuspended in chilled PBS and then incubated with 5 µl of the PI and Annexin V-FITC kit reagents for 15 min at room temperature in the dark before being subjected to flow cytometry. Each experiment was performed in triplicate.

### Hoechst 33258 staining

Cells were seeded in a six-well plate at an initial density of 6 × 10^5^ cells/well and treated with different concentrations of TMJ-12 (0, 8, and 16 µM) for 48 h. The cells were then collected and washed twice with PBS, and chromatin condensation was detected using the Hoechst 33258 Staining Kit (Beyotime). Stained nuclei were immediately photographed under a fluorescence microscope.

### Detection of the mitochondrial membrane potential

The mitochondrial membrane potential (ΔΨm) of the cells was measured using the JC-1 assay (Beyotime). BPH-1 cells were seeded in six-well plates (3 × 10^5^ cells/well) and incubated at 37°C for 24 h. Next, the cells were treated with different concentrations of TMJ-12 (0, 8, and 16 µM) for 48 h, after which, the cells were collected and incubated with 10 mM JC-1 in the dark at 37°C for 30 min. Finally, JC-1 fluorescence was measured using the flurescence microscope (BD Biosciences, NJ, U.S.A.).

### Western blotting

After the BPH-1 cells had been treated with TMJ-12 (0, 8, 16 μM) for 24 h, the cells were collected, washed twice with ice-cold PBS, and lysed in RIPA buffer containing 1% (w/v) of a PMSF protease inhibitor (Solarbio) at 4°C for 30 min. Cell debris was removed by centrifugation at 12000 rpm for 20 min at 4°C, and the protein concentrations were determined with the BCA Protein Assay Kit (Solarbio). Proteins were separated by 10% sodium dodecyl sulfate/polyacrylamide gel electrophoresis (SDS/PAGE) and transferred by Western blotting to nitrocellulose membranes. The membranes were blocked with 5% non-fat milk for 2 h, incubated overnight with primary antibodies at 4°C, and then incubated with a fluorescently labeled goat anti-rabbit IgG secondary antibody (Cell Signaling Technology, Danvers, MA, U.S.A.) for 2 h at room temperature. GAPDH was used as an internal control. Immunoreactive proteins were visualized with the Odyssey Infrared Imaging System (LI-COR Biotechnology, Lincoln, NE, U.S.A.) and densitometry analyses of the western blot bands were performed with ImageJ software.

### Molecular docking

The protein structure of PTEN (PDB ID: 1D5R) required for docking was obtained from the PDB database (https://www.rcsb.org/). AutoDockTools 1.5.6 software [23] was used to remove the water molecules, perform hydrogenation, and calculate the charge of the protein. The structure was then saved in the pdbqt format. Subsequently, the Autodock Vina [24] program was used for molecular docking with the parameters set at num_modes = 10, energy_range = 4, and exhaustiveness = 100.

### Statistical analysis

All data are expressed as the mean ± standard deviation (SD) of at least three independent experiments. Each experiment was repeated in triplicate. Statistical analyses were performed with Student’s *t* test or one-way analysis of variance. Significance levels were defined as follows: not significant (ns), *P<0.05; **P<0.01; ***P<0.001.

## Results

### Effect of TMJ-12 on BPH-1 cell viability

To investigate the effect of TMJ-12 on the viability of the BPH-1 cells, the MTT assay was performed on BPH-1 cells treated with increasing concentrations of TMJ-12 (0–16 μM) for 24, 48, or 72 h (Figure 1B). TMJ-12 was found to reduce BPH-1 cell viability in both dose- and time-dependent manner. The IC_50_ of TMJ-12 toward the BPH-1 cells was 8.02 μM after 72 h of incubation (Figure 1C). TMJ-12 treatment was also observed to induce noticeable morphological changes in these cells (Supplementary Figure S1). The colony formation assay was used to further assess the effects of TMJ-12 on BPH-1 cell proliferation (Figure 1D,E). Increasing concentrations of TMJ-12 (0–16 μM) were found to reduce the rate of colony formation of the BPH-1 cells in a dose-dependent manner.

### TMJ-12 induces G_2_/M phase cell cycle arrest of BPH-1 cells

**Figure 2:**
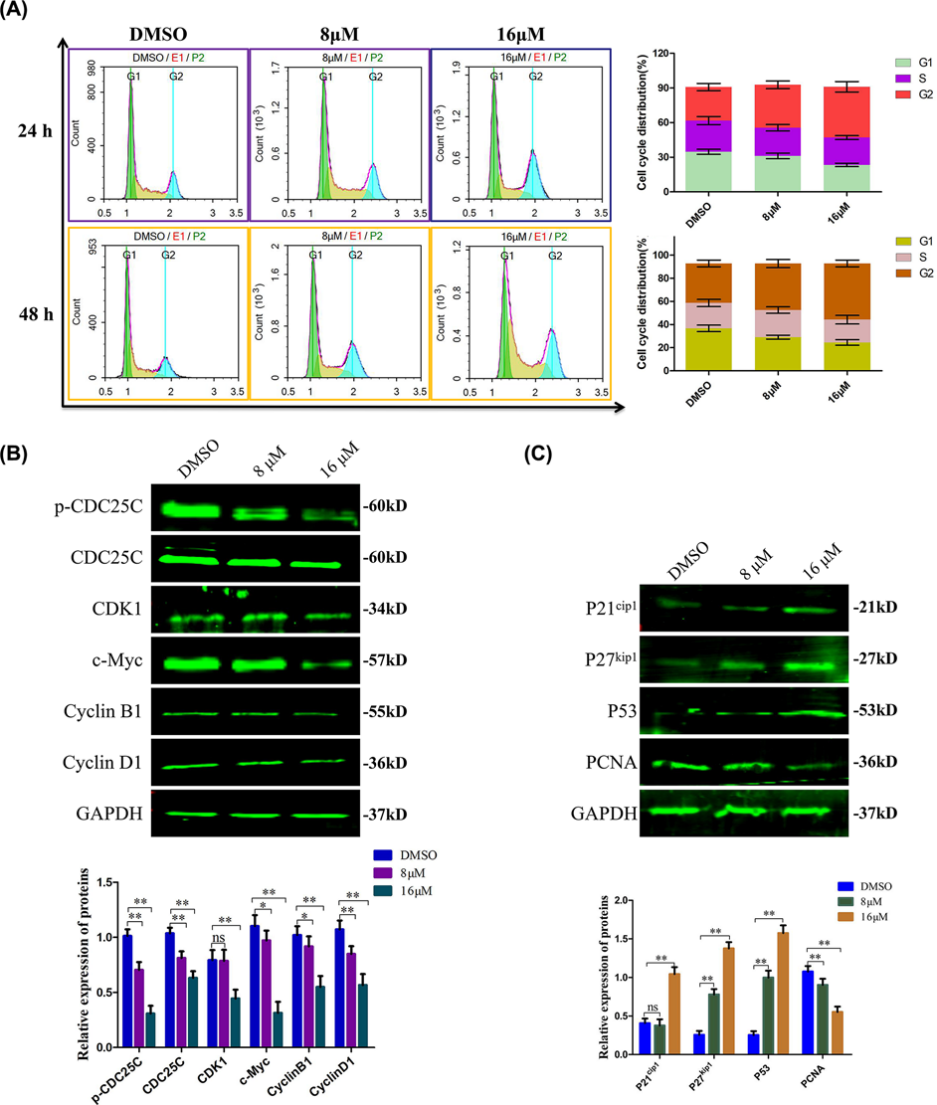
TMJ-12 induces G_2_/M phase cell cycle arrest of BPH-1 cells (**A**) The cell cycle phase distribution of BPH-1 cells treated with various concentrations of TMJ-12 for 24 or 48 h was determined using PI. The percentage of the cell cycle distribution at the G_1_, S, and G_2_ phases are shown on the right as the means ± SD, *n*=3. (**B,C**) BPH-1 cells were exposed to TMJ-12 (8 or 16 μM) for 24 h, and the protein expression levels of core factors associated with cell cycle progression were measured by Western blotting. GAPDH was used as the loading control. Data represent the mean ± SD, *n*=3. *P<0.05, **P<0.01 vs. the control group; ns, not significant.

Since TMJ-12 inhibited BPH-1 cells proliferation, we further examined whether TMJ-12 effects BPH-1 on cell cycle progression. The treatment of BPH-1 cells with various concentrations of TMJ-12 (8 or 16 µM) for 24 and 48 h respectively, and cell cycle distribution was determined by PI staining using flow cytometry. With the increase in TMJ-12 concentration and the prolongation of treatment time, the result indicated that TMJ-12 treatment led to a marked increase in cells at G_2_/M phase (Figure 2A).

To understand the underlying molecular events, we examined numerous cell cycle regulators. Western blot analyses revealed that the cell cycle family proteins; CDK1, CyclinB1, CyclinD1, c-Myc, and the CDC25C were consistently declined in treatment with TMJ-12 cells (Figure 2B). One of most important protein was CDC25C, which was critical for the G_2_ checkpoint, while the P27^kip^, P21^cip^, and P53 were substantially elevated. In addition, proliferating cell nuclear antigen (PCNA), a downstream marker of proliferation, was remarkably down-regulated in TMJ-12-treated cells (Figure 2C). Thus, our result showed that effect of TMJ-12 inhibited BPH-1 cell viability was related to cell cycle arrest.

### TMJ-12 induces BPH-1 cell apoptosis via the mitochondrial apoptotic pathway

**Figure 3:**
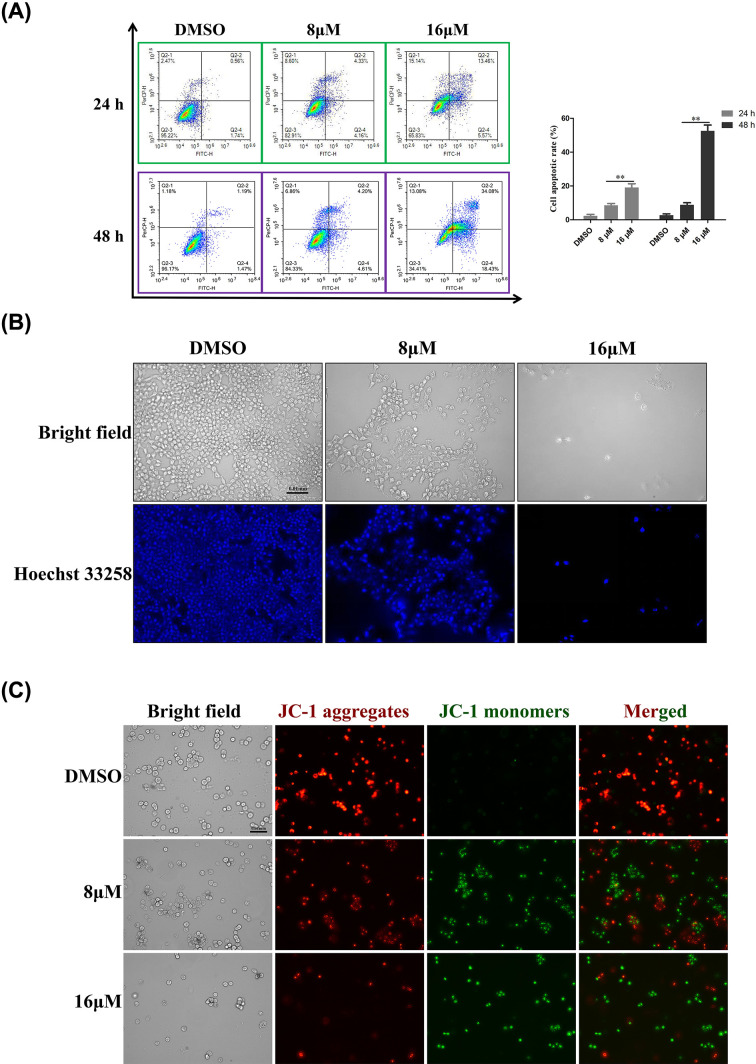
TMJ-12 induces the apoptosis of BPH-1 cells via the mitochondrial apoptotic pathway (**A**) BPH-1 cells were treated with TMJ-12 for 24 or 48 h, stained with Annexin V-FITC and PI, and then analyzed by flow cytometry. The bar plot indicates the percentage of total apoptosis in each treatment group. Data represent the mean ± SD, *n*=3. **P<0.01 vs. the control group. (**B**) BPH-1 cells were stained with Hoechst 33258 and examined under a fluorescence microscope (magnification, ×200; scale bar, 0.01 mm). (**C**) BPH-1 cells were treated with TMJ-12 for 24 h, stained with the fluorescent dye, JC-1, and the ΔΨm was then examined by fluorescence microscopy (magnification, ×200; scale bar, 0.01 mm). The distributions of the red (JC-1 aggregates) versus green (JC-1 monomers) fluorescence in the BPH-1 cells are shown.

Apoptosis is a form of programmed cell death and mitochondrial changes are considered to be a hallmark of apoptosis [25]. Here, double-staining with Annexin V-FITC and PI was employed to analyze the rate of apoptosis of the BPH-1 cells following TMJ-12 treatment, while Hoechst 33258 staining was used to visualize the cell death caused by TMJ-12. Treatment of the BPH-1 cells with TMJ-12 (8 or 16 µM) for 24 or 48 h was shown to induce apoptosis (Figure 3A,B), particularly in the high-dose groups. Following 48 h of treatment with TMJ-12 at 16 μM, 52.51% of the BPH-1 cells were observed to have undergone apoptosis.

Next, the JC-1 staining assay was used to investigate whether TMJ-12-induced apoptosis is mediated by the mitochondrial pathway. Compared with the untreated cells, treatment with 8 or 16 μM TMJ-12 for 24 h caused a dose-dependent increase in the JC-1 aggregate (red) vs*.* monomer (green) fluorescence ratio of the BPH-1 cells (Figure 3C). These results indicated that TMJ-12 caused mitochondria dysfunction and triggered the mitochondrial apoptotic pathway.

**Figure 4:**
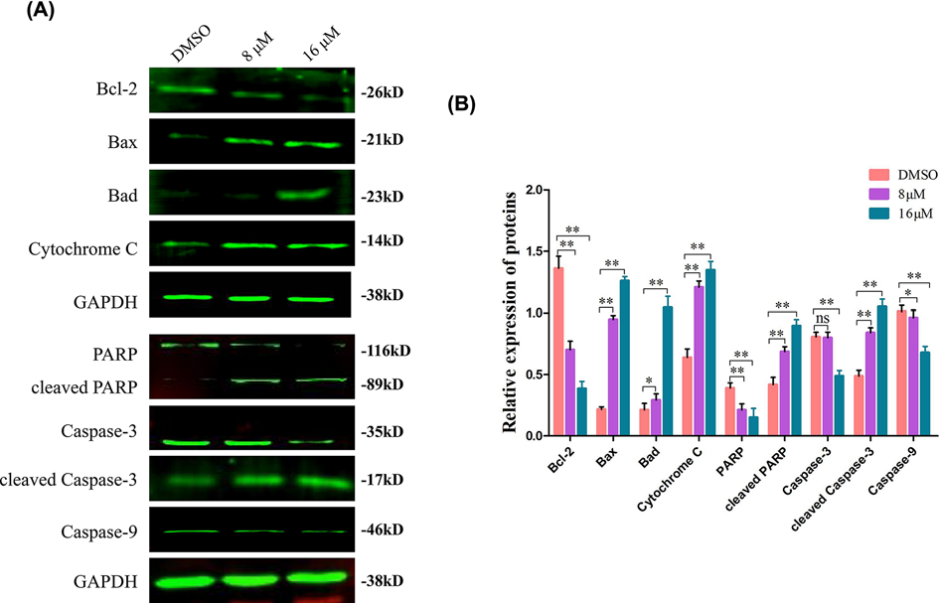
Effect of TMJ-12 on the expression of proteins involved in the apoptosis of BPH-1 cells (**A,B**) BPH-1 cells were treated with TMJ-12 for 24 h and then subjected to Western blot analysis using antibodies against various known apoptotic factors. GAPDH was used as the loading control. Data represent the mean ± SD, *n*=3. *P<0.05, **P<0.01 vs. the control group.

Western blot analysis of several apoptosis- and cell cycle-related proteins revealed that TMJ-12 down-regulated the expression of Caspase-3 but increased the cleavage (activation) of this caspase, thereby inducing the cleavage of poly (ADP-ribose) polymerase (PARP) (Figure 4A,B). The ratio of Bax/Bcl-2 was markedly increased in the TMJ-12-treated cells, and the level of cytochrome *c* in the mitochondria of these cells also increased.

### TMJ-12 inhibits activation of the PI3K/AKT signaling pathway

Several studies have demonstrated that the PI3K/AKT signaling pathway is closely associated with the cell cycle and plays a critical role in controlling cell survival/death. Abnormal activation of the PI3K pathway results in disturbances in the control of cell growth and survival, which can contribute toward cells acquiring a competitive growth advantage, metastatic competence, and, frequently, therapeutic resistance [26]. Thus, we next investigated whether TMJ-12 activated the PI3K/AKT pathway.

**Figure 5:**
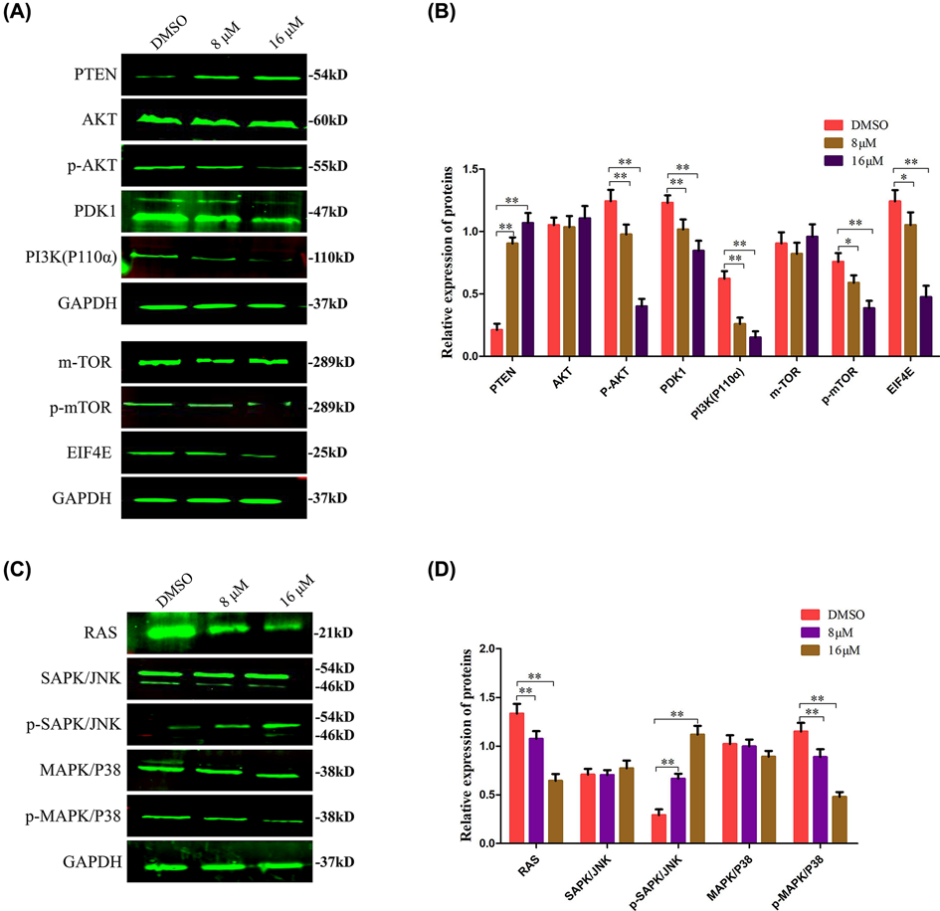
The inhibitory effect of TMJ-12 on BPH-1 cells is mediated by activation of the PI3K/AKT signaling pathway (**A–D**) BPH-1 cells were treated with various concentrations of TMJ-12 for 24 h, and the protein expression levels of various factors along the PI3K/AKT signaling pathway were analyzed by Western blotting. GAPDH was used as the loading control. Data represent the mean ± SD, *n*=3. *P<0.05, **P<0.01 vs. the control group.

Western blot analysis of BPH-1 cells indicated that treatment with TMJ-12 reduced the expression levels of PI3K (subunit p110α) and its downstream regulator PDK1, as well as the phosphorylation of AKT at Ser^473^ (Figure 5A,B). Diminished levels of phosphorylation at Ser^2448^ of the mTOR protein was also observed. Further investigation indicated that TMJ-12 treatment inhibited the expression of EIF4E, the phosphorylation target of mTOR, whereas the expression of PTEN increased. These data together imply that TMJ-12 may inhibit AKT through the activation of PTEN and the inhibition of PDK1 and PI3K.

The expression and phosphorylation levels of MAPK/P38 and SAPK/JNK, as components of the side pathway of PI3K/AKT signaling [27], were also determined, as these play crucial roles in regulating apoptosis (Figure 5C,D). The results showed that the phosphorylation levels of MAPK/P38 decreased while the protein levels of SAPK/JNK increased in a time-dependent manner.

**Figure 6:**
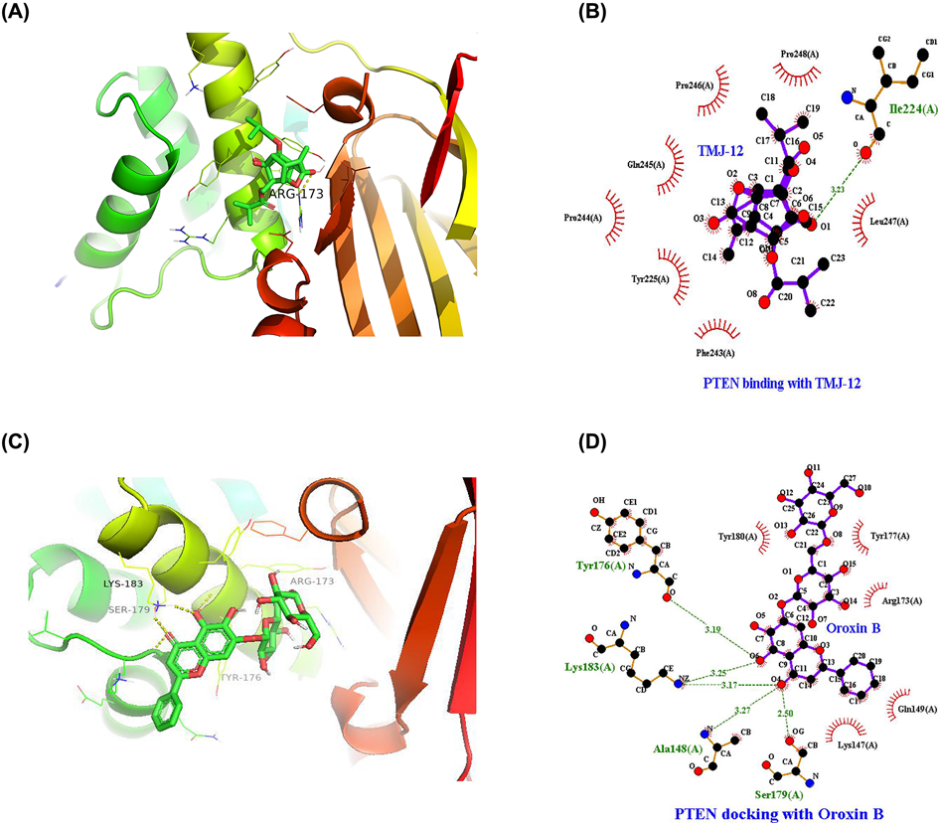
Computational docking analysis of TMJ-12 with the PTEN protein (**A,B**) The binding conformation of TMJ-12 in the active side of PTEN and the Schematic diagram representing the PTEN-binding site and proximate affinity of TMJ-12. (**C,D**) The binding conformation of Otoxin B in the active side of PTEN and Schematic diagram representing the PTEN-binding site and proximate affinity of Oxtoxin B. Black dots: carbon atoms; blue dots: nitrogen atoms; red dots: oxygen atoms; green dotted lines: hydrogen bonds; red combs: amino acid residues.

Finally, unbiased blind docking was used to predict the region involved in binding between TMJ-12 and PTEN. For this purpose, all known DNA-binding domain structures of PTEN as identified by X-ray crystallography (PDB ID: 1D5R) were used. The docking of TMJ-12 with ID5R showed a low binding energy of −9.23 kJ/mol (Figure 6A,B). Both compounds formed similar Van der Waals interactions with the residuals including Tyr^176^, Pro^169^, Arg^173^, Tyr^180^, and Tyr^177^, and the docking results indicated that TMJ-12 can hydrogen bond to Arg^173^ of PTEN. Also, we used Oroxin B which is the activator of PTEN as a positive control [28] (Figure 6C,D). In comparison to Oroxin B, TMJ-12 formed more hydrogen bonds with PTEN. These data indicated that TMJ-12 enhanced the activity of PTEN. Thus, the docking analysis findings suggest that TMJ-12 can directly bind with PTEN and may thereby inhibit the PI3K/AKT signaling pathway.

## Discussion

Natural products have been a rich source of compounds for drug discovery, and continue to enter clinical trials as anticancer and antimicrobial agents [29]. They also exhibit diverse structures that correspond to the diversity of their biological functions and binding partners [30]. Our previous study found that TMJ-12, a germacrane sesquiterpenoid extracted from *C. cernuum*, inhibits the proliferation of the cervical cancer cell line, HeLa, and the liver cancer cell line, HepG2 [31]. In the present study, TMJ-12 was tested on the BPH-1.

In the present study, we focused on the inhibitory effect of TMJ-12 on the proliferation of BPH-1 cells and explored the mechanisms involved. Both the MTT assay and colony formation assay demonstrated that TMJ-12 has a strong inhibitory effect on the proliferation of BPH-1 cells in a time- and dose-dependent manner. Further, flow cytometry assays showed that TMJ-12 can block the growth of BPH-1 cells in the G_2_/M phase and promote BPH-1 cell apoptosis.

Previous studies have demonstrated that cell apoptosis occurs mainly through the intrinsic mitochondrial pathway and the extrinsic death receptor pathway. The mitochondrial pathway of apoptosis is known to depend on the activation of caspases, which ultimately results in cell death characterized by cellular shrinkage, chromatin condensation, and membrane blebbing [32]. This pathway is triggered by cellular stress or developmental cues that cause mitochondrial damage, leading to the release of Cytochrome *c*, apoptosis-inducing factor, and procaspase-9 into the cytosol and the formation of a complex termed the apoptosome [33,34]. Our data showed that TMJ-12 likely induces mitochondria-mediated apoptosis in BPH-1 cells, as treatment with TMJ-12 resulted in reduced ΔΨm, increased Bax/Bcl-2 expression ratio, increased level of Cytochrome *c* released into the cytosol, activation of Caspase-9 (an initiator caspase) such that it could enter the cell and form a complex termed the apoptosome, followed by the release of Caspase-3 and cleavage of PARP.

Many antitumor agents inhibit cell replication by blocking the cell cycle at specific checkpoints. CDC25C is one of the key proteins regulating cell entry to the G_2_/M phase and providing positive feedback to regulate cell mitosis, and CDC25C is known to be activated by CDK1 and Cyclin B1 [35,36]. Our study showed a significant decrease in CDC25C, p-CDC25C, CDK2, Cyclin B1, and c-Myc, along with an increase in P53, P21^Cip1^, and P27^Kip1^ in BPH-1 cells when treated with TMJ-12. These findings indicated that TMJ-12 may block the growth of BPH-1 cells in the G_2_/M phase.

**Figure 7:**
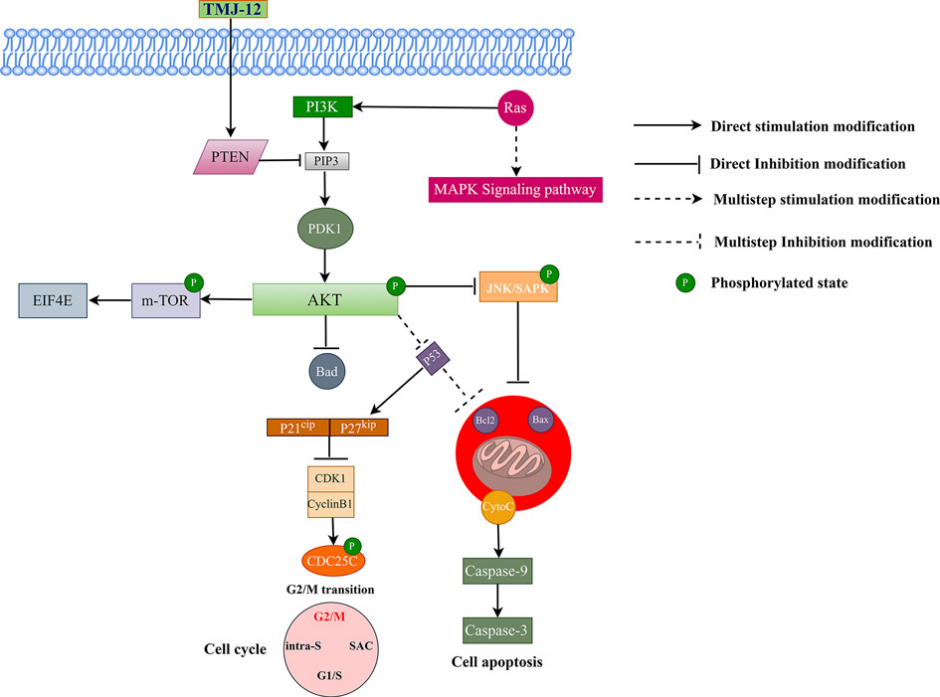
Schematic diagram depicting the effect of TMJ-12 on the PI3K/AKT signaling pathway leading to the induction of apoptosis and cell cycle arrest in BPH-1 cells

The effect of TMJ-12 on the PI3K/AKT signaling pathway was also investigated. The PI3K pathway regulates various cellular processes, such as proliferation, growth, and apoptosis. In the present study, we observed that TMJ-12 induced the expression of PTEN and inhibited PI3K/AKT phosphorylation, suggesting that TMJ-12 may be involved in cell cycle arrest and apoptosis (Figure 7). To further investigate whether TMJ-12 affects the PI3K/AKT pathway by regulating PTEN, molecular docking was applied and TMJ-12 was found to exhibit strong affinity toward PTEN *in silico*.

In conclusion, the present study demonstrated that TMJ-12, which is a compound extracted from *C. cernuum*, offers broad-spectrum antitumor activity against the BPH-1. TMJ-12 was shown to promote BPH-1 cell apoptosis and cell cycle arrest at G_2_/M. In addition, several key factors along the PI3K/AKT pathway were found to exhibit significantly altered expression following TMJ-12 treatment, which would have resulted in the increased rates of cell apoptosis observed. Overall, our findings suggest that TMJ-12 may be a promising lead compound for the treatment of BPH.

## Supplementary Material

Supplementary Figure S1

Supplementary Data S1

## Data Availability

All the data can be obtained from the corresponding authors.

## Abbreviations

AKT: Protein kinase B
Annexin V-FITC: Annexin V-fluorescein isothiocyanate
BPH: Benign prostatic hyperplasia
BPH-1: Benign prostatic hyperplasia cell line
CDK: Cyclin-dependent kinase 1/2
DHT: Dihydrotestostrone
FBS: Fetal bovine serum
MTT: 3-(4,5-dimethylthiazol-2-yl)-2,5-diphenyltetrazolium bromide
PARP: Poly (ADP-ribose) polymerase
PBS: Phosphate-buffered saline
PCNA: Proliferating cell nuclear antigen
PDK1: 3-phosphoinositide-dependent protein kinase 1
PI: Propidium iodide
PI3K: Phosphatidylinositol 3-kinase
PTEN: Phosphatase and tensin homolog deleted on chromosome 10
SAPK: Stress-actived protein kinase
ΔΨm: Mitochondrial membrane potential
