# Supplementary material for: Incaspitolide A extracted from Carpesium cernuum induces apoptosis in vitro via the PI3K/AKT pathway in benign prostatic hyperplasia
Source: Biosci Rep. 2021 Jun 21;41(6):BSR20210477. doi: 10.1042/BSR20210477 (PMC8220449; doi:10.1042/BSR20210477)
Supplement: Supplementary Figure S1 [file BSR-2021-0477_supp.pdf]

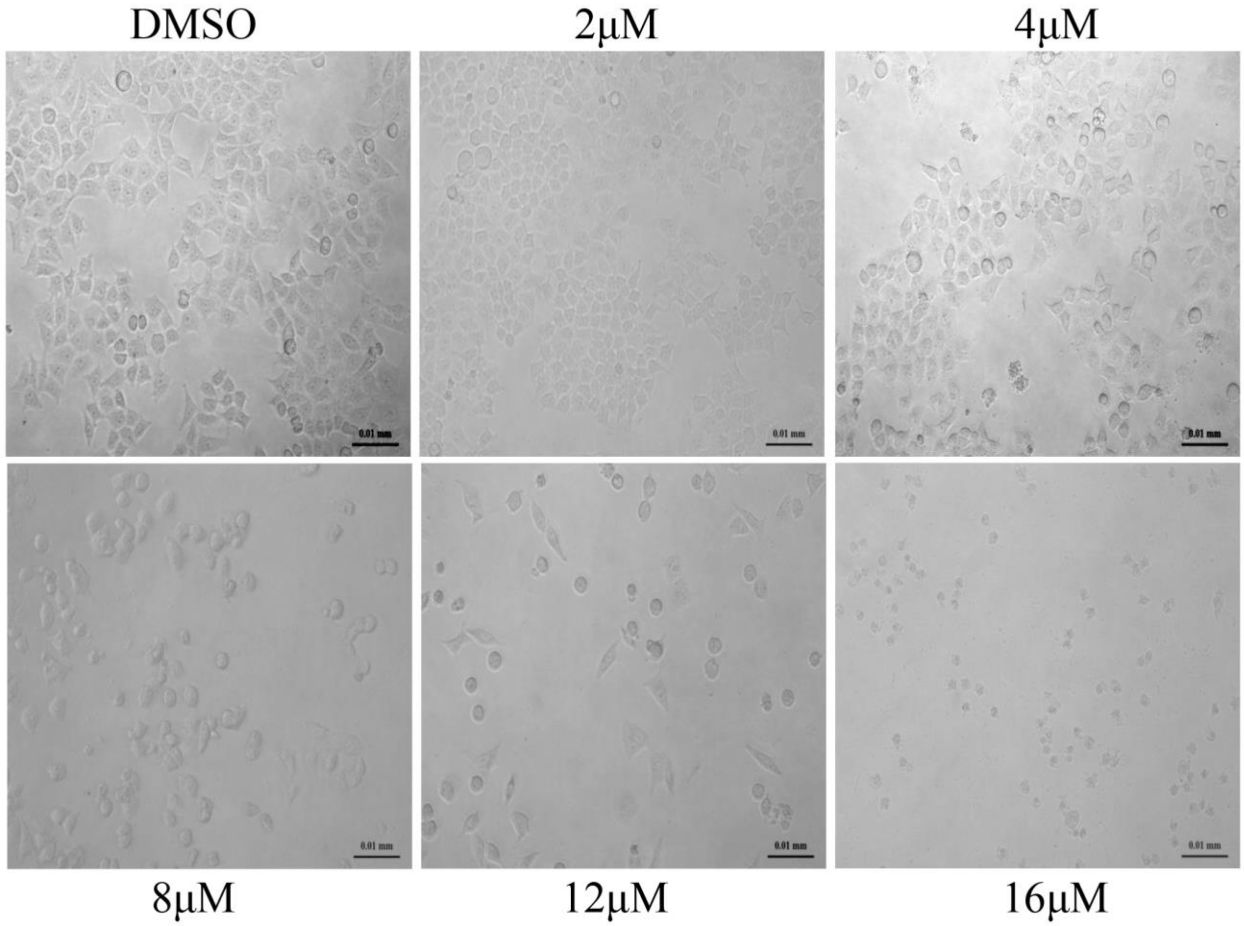

Supplementary Figure 1. Representative image of BPH-1 cells treated with TMJ-12 for 48 h (magnification, ×200; scale bar, 0.01 mm), TMJ-12 inhibited the cell viability and proliferation of the BPH-1 cells.
