# Supplementary material for: Incaspitolide A extracted from Carpesium cernuum induces apoptosis in vitro via the PI3K/AKT pathway in benign prostatic hyperplasia
Source: Biosci Rep. 2021 Jun 21;41(6):BSR20210477. doi: 10.1042/BSR20210477 (PMC8220449; doi:10.1042/BSR20210477)

Supplementary data

Purity analysis of incaspitolide A.

The HPLC of incaspitolide A isolated from *Carpesium cernuum* L.

1.00 mg/ml; 210 mm-254 mm; Chromatographic methanol: H_2O = 65:35; 1.00 ml/min Purity: 98.6536%

	peak #	retention time	Concentration	area	height	peak start	peak start	peak end	area%
Control	1	3.834	0.00000	237256	8692	S	3.217	7.475	100.0000
T12	1	3.831	0.00000	419903	17414	SV	3.433	6.075	11.0329
	2	8.417	0.00000	3334778	170592	SV	7.792	10.775	87.6207
	3	18.989	0.00000	51243	1206	V	18.442	19.617	1.3464
									98.6536

^{1}H NMR spectrum of incaspitolide A in $CDCl_3$ (INOVA 400 MHz).

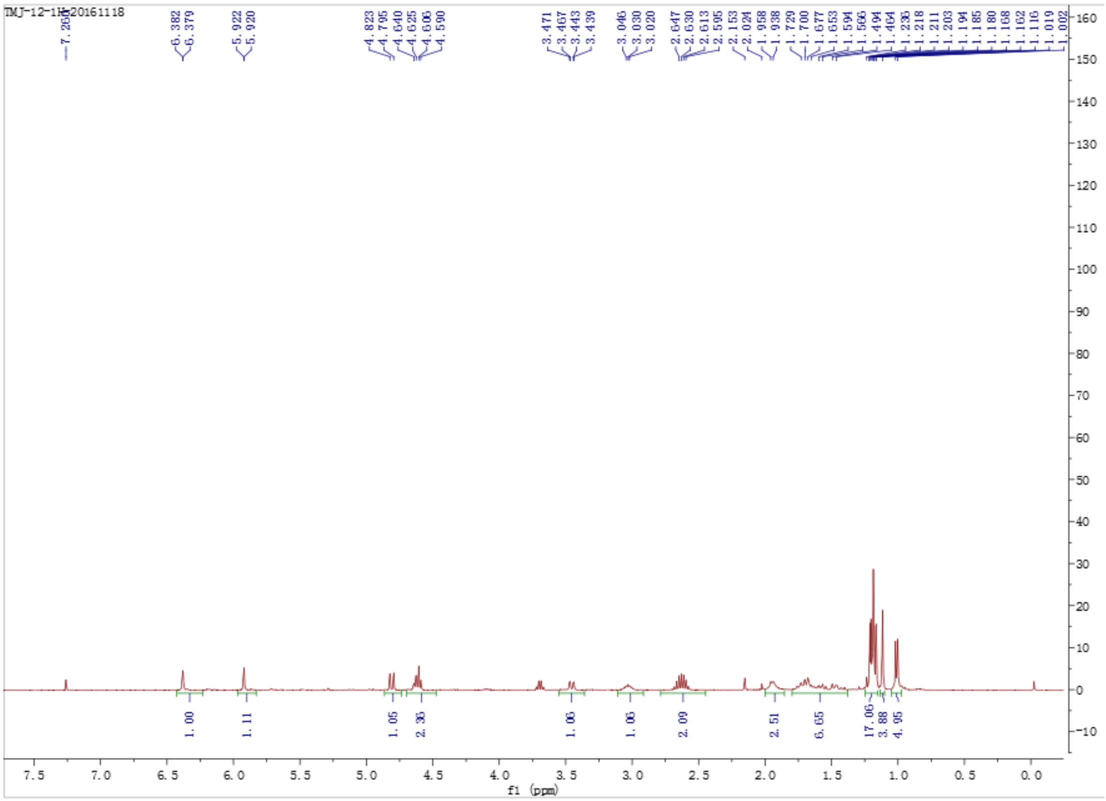

^{13}C NMR spectrum of incaspitolide A in $CDCl_3$ (INOVA 400 MHz).

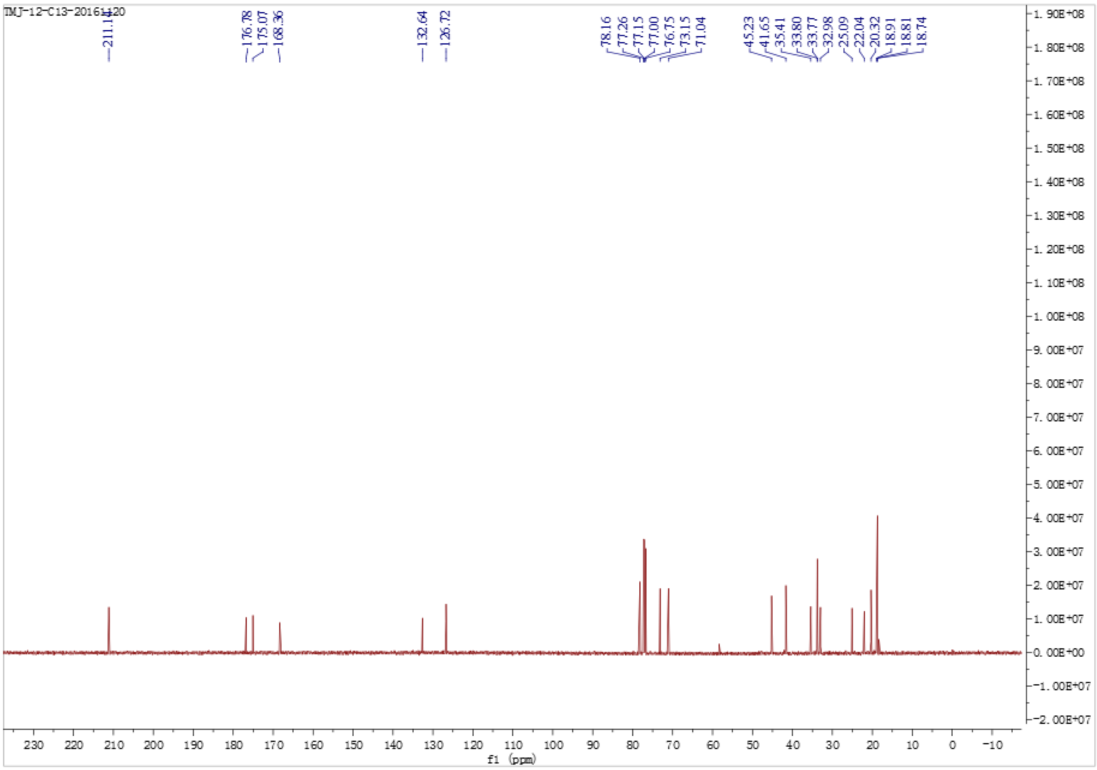

Supplement: Supplementary Data S1 [file BSR-2021-0477_supp1.pdf]
